# Choroidal macrovessels: multimodal imaging findings and review of the literature

**DOI:** 10.1136/bjophthalmol-2020-318095

**Published:** 2021-01-04

**Authors:** Beatrice Gallo, Samantha R de Silva, Omar A Mahroo, Zubin Saihan, Praveen J Patel, Jonathan G Dowler, Carlos Pavesio, Pearse A Keane, Adnan Tufail, Mandeep S Sagoo

**Affiliations:** 1 Ocular Oncology Service, Moorfields Eye Hospital NHS Foundation Trust, London, UK; 2 Medical Retina Service, Moorfields Eye Hospital NHS Foundation Trust, London, UK; 3 Institute of Ophthalmology, University College London, London, UK; 4 NIHR Biomedical Research Centre for Ophthalmology, Moorfields Eye Hospital and University College London Institute of Ophthalmology, London, UK

**Keywords:** choroid, diagnostic tests/investigation, retina

## Abstract

**Background/aims:**

To describe clinical and multimodal imaging features in a cohort of choroidal macrovessels.

**Methods:**

Demographics and multimodal imaging features of 16 eyes of 13 patients with choroidal macrovessels were reviewed. The multimodal imaging included colour fundus photography, fundus autofluorescence (FAF), spectral domain enhanced depth imaging optical coherence tomography (OCT), en face OCT, OCT-angiography (OCT-A), B-scan ultrasonography (US), fluorescein angiography (FFA) and indocyanine green angiography (ICGA).

**Results:**

Three patients had bilateral involvement. On colour fundus photography, three patterns were evident (a clearly visible orange-red vessel; a track of pigmentary changes; spots of mild pigmentary changes). Vessel orientation was horizontal (11 eyes), oblique (4 eyes) or vertical (1 eye). In 2 eyes, the vessel was extra-macular. OCT in all cases showed a hyporeflective choroidal area with posterior shadowing and elevation of the overlying retina. Subretinal fluid was present in 4 eyes. FAF (12 eyes) was normal (7 eyes) or showed a hypofluorescent/hyperfluorescent track (4 eyes) or linear hyperautofluorescence (1 eye). En-face OCT (2 eyes) revealed the course of the macrovessel at the level of choroid and choriocapillaris. On OCT-A (2 eyes) the vessel had a reflectivity similar to surrounding vessels but larger diameter. B-scan US (8 eyes) showed a nodular hypoechogenic lesion. FFA (5 eyes) showed early focal hyperfluorescence (4 eyes) not increasing in later phases, or was normal (1 eye). ICGA (6 eyes) showed early hyperfluorescence of the vessel.

**Conclusions:**

Choroidal macrovessels can mimic other entities, leading to underdiagnosis. Appreciating relevant features on different imaging modalities will aid a correct diagnosis.

## Introduction

A choroidal macrovessel (CM) is a rare anatomical abnormality, reported in the literature in fewer than a dozen reports,^1–12^ consisting of a vessel in the choroid with a larger diameter and a more pronounced tortuosity than the surrounding vasculature. In the first report in 2011, Lima *et al* described such an anomalous choroidal vessel extending from the temporal paramacular area to the temporal periphery.^1^ The largest series to date comprises two cases occurring in two white females aged 55 and 68 years old.^2^

The purpose of our study is to describe the clinical features of CM in a larger series by using a multimodal imaging approach and to compare our findings to those reported in the literature. In the previous reported cases, the majority are isolated case reports,^1–12^ including two earlier publications from our department which are also included in this updated report.^3 4^

The pathogenesis of CM is not understood, and it is unclear whether it is a congenital or an acquired condition, and if it is associated with systemic vascular abnormalities. In the previously reported cases CM was located in the temporal macula, had a horizontal orientation, a diameter ranging between 200 and 300 µm and a length between 6 and 11 mm. Given its rarity, CM is often not recognised or can be misdiagnosed, potentially masquerading as other conditions, like parasitic infestations,^5 6^ choroidal tumours,^2^ vortex vein ampullae, retino-choroidal anastomosis,^7^ diffuse unilateral subacute neuroretinitis,^1^ anomalous posterior ciliary vessels and congenital retinal macrovessels.^1^

## Subjects and methods

Electronic medical records for all patients attending the Ocular Oncology and Medical Retina Services of Moorfields Eye Hospital between January 2015 and January 2020 were searched for the term ‘choroidal macrovessel ’.

Data collected for each patient included sex, age, ethnicity, disease laterality, symptoms, ocular and systemic diseases. All patients underwent a comprehensive ophthalmological examination including best-corrected visual acuity (expressed in Snellen or logarithm of the Minimum Angle of Resolution, logMAR), intraocular pressure, slit lamp biomicroscopy and dilated funduscopy. Multimodal imaging used to study the CM included colour fundus photography (Topcon Corporation, Tokyo, Japan), ultrawide field pseudocolour fundus photography (Optos plc, Dunfermline, UK), fundus autofluorescence (FAF) (Optos, or Spectralis, Heidelberg Engineering, Heidelberg, Germany), near infrared reflectance (Spectralis) images, fundus fluorescein angiography (FFA) and indocyanine green angiography (ICGA) (Optos or Spectralis), spectral domain enhanced depth imaging (EDI) optical coherence tomography (OCT) (Spectralis) and swept source OCT (Topcon Corporation), OCT-Angiography (OCT-A) and OCT en face (Zeiss, Oberkochen, Germany), ocular ultrasound (US, Siemens Healthcare, Munich, Germany). Some images were acquired using the ‘multicolour’ setting of the Heidelberg Spectralis, combining red, green and blue wavelength reflectance imaging.

## Results

We identified 16 eyes of 13 patients with CM that fulfilled the criteria for this study. Patient demographics, disease laterality, disease location, symptoms, systemic and ocular comorbidities are listed in table 1. Colour (or pseudocolour/multicolour) fundus images and OCT were obtained from all 16 eyes, FAF from 12 eyes, US from 8 eyes, ICGA from 6 eyes, FFA from 5 eyes, OCT-A and en face OCT from 2 eyes.

**Table 1:** Summary of patient demographics, CM laterality and location, BCVA, symptoms, systemic and ocular comorbidities associated to CM in the study cohort

Feature	N (%)	Systemic comorbidities	n=13 (%)
Sex		Hypertension	5 (39)
Female	8 (62)	Diabetes mellitus	3 (23)
Male	5 (38)	Hypothyroidism	1 (8)
Age (years)		Peripheral vascular disease	1 (8)
Mean	66	Heart disease	1 (8)
Median	67	Hyperlipidaemia	1 (8)
Range	27–92	Benign prostatic hypertrophy	1 (8)
Ethnicity		Vertigo	1 (8)
Caucasian	4 (31)	Breast cancer	1 (8)
African	2 (15)	**Ocular co-morbidities**	**n=16 (%)**
Afro-caribbean	1 (8)		
N/A	7 (46)	Glaucoma	4 (25)
Laterality		Age-related macular degeneration	3 (19)
RE	2 (15)	Non-proliferative diabetic retinopathy	2 (13)
LE	8 (62)	Retinal detachment	1 (6)
BE	3 (23)	Central retinal vein occlusion	1 (6)
Location		Branch retinal vein occlusion	1 (6)
Macular	14 (88)		
Extramacular	2 (12)		
Location relative to fovea			
Temporal	7 (50)		
Supero-temporal	4 (29)		
Supero-nasal	1 (7)		
Nasal and temporal	1 (7)		
Superior and inferior	1 (7)		
BCVA (Snellen, logMAR)			
Mean	6/7.5 (0.10 logMAR)		
Range	6/6-6/60 (0–1 logMAR)		
Symptoms			
Yes	2 (13)		
No	14 (87)		

BCVA, best corrected visual acuity; BE, both eyes; CM, choroidal macrovessel; LE, left eye; logMAR, logarithm of the minimum angle of resolution; N/A, not available; RE, right eye.

There were 5 males and 8 females, and the mean patient age was 66 years (range 27–92; median 67 years). The recorded ethnicity was Caucasian in 4 patients (31%), African in 2 (15%), African Caribbean in 1 (8%) and unknown in 7 (46%). The left eye was affected in 8 patients (62%), the right in 2 patients (15%) and both in 3 patients (23%). Symptoms, of decreased central vision and metamorphopsia, were present in only 2 eyes (13%) with a best corrected Snellen visual acuity of 6/60 (1.0 logMAR) and 6/18 (0.48 logMAR), respectively. The remaining 14 eyes of 11 patients were asymptomatic with a mean best corrected Snellen visual acuity of 6/7.5 (0.10 logMAR).

Recorded systemic diseases included hypertension in 5 patients (39%), diabetes mellitus in 3 patients (23%), hypothyroidism in 1 patient (8%), peripheral vascular disease in 1 patient (8%), heart disease in 1 patient (8%), hyperlipidaemia in 1 patient (8%), benign prostatic hypertrophy in 1 patient (8%), vertigo in 1 patient (8%) and breast cancer in 1 patient (8%).

Ocular comorbidities included glaucoma in 4 eyes (25%) from 3 patients, age-related macular degeneration (AMD) in 3 eyes (19%) from 2 patients, non-proliferative diabetic retinopathy in 2 eyes (13%) from 1 patient, retinal detachment in 1 eye (6%), central retinal vein occlusion in 1 eye (6%), branch retinal vein occlusion in 1 eye (6%). One patient with bilateral CMs had a history of retinal detachment, central and branch retinal vein occlusion in one eye and of neovascular AMD (nAMD) in the other eye. Data on refractive error or axial length were not available.

**Figure 1:**
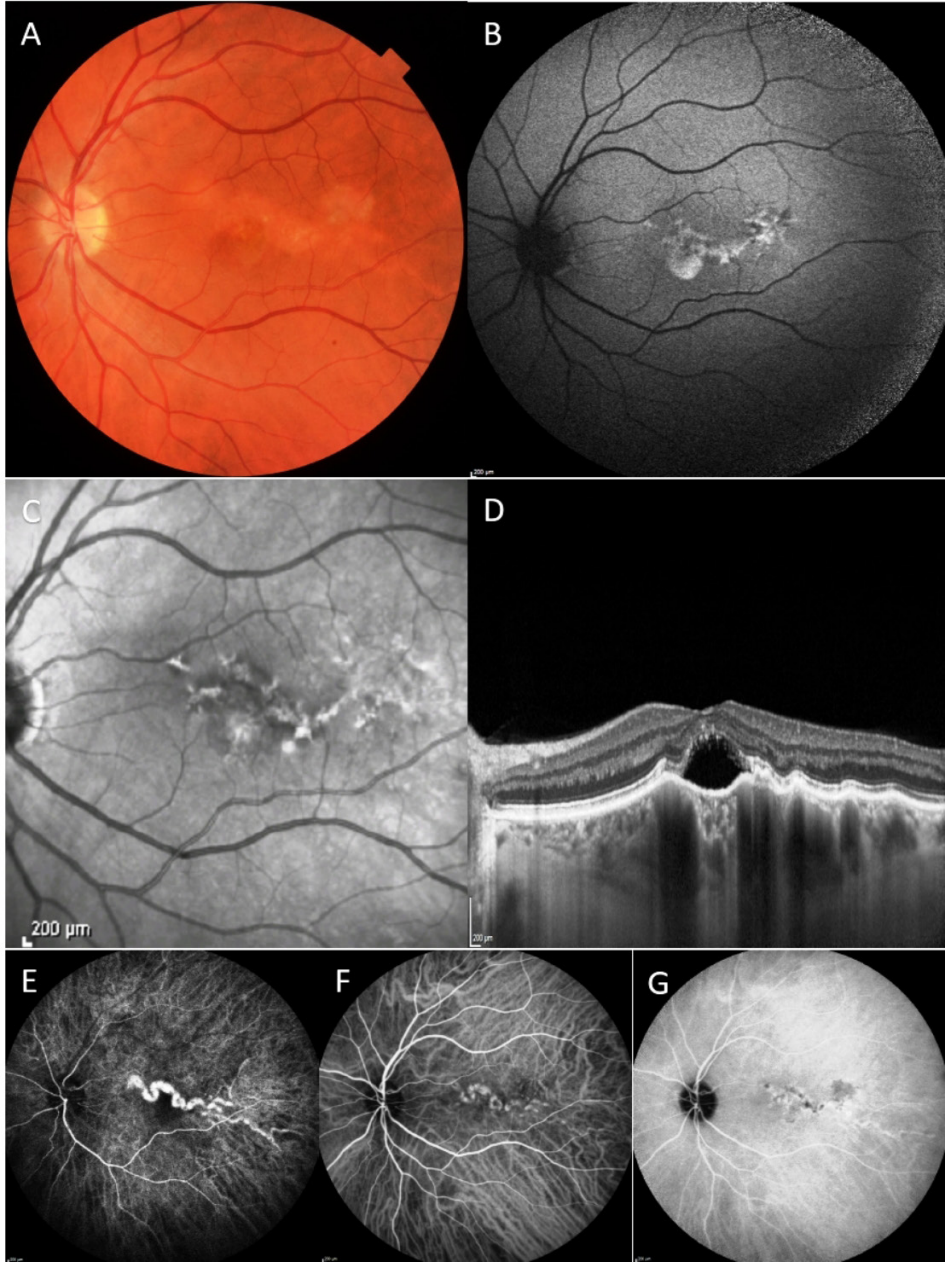
Colour fundus photograph, FAF, near-infrared image, EDI-OCT and ICGA of a patient with type I CM. (A) Colour photograph shows a serpiginous lesion extending horizontally from the macula towards the temporal periphery. (B) FAF shows a hypo/hyperautofluorescent pattern in correspondence of the vessel. (C) Near-infrared image showing hyper-reflective and hyporeflective spots in a track-like fashion. (D) EDI-OCT through the macula shows the foveal cm. (E) ICGA shows the early hyperfluorescence and the serpiginous shape of the vessel. (F, G) ICGA in the later phases shows a reduced fluorescence similar to the surrounding vessels with hypofluorescent spots. EDI, enhanced depth imaging; FAF, fundus autofluorescence; ICGA, indocyanine green angiography; OCT, optical coherence tomography.

**Figure 2:**
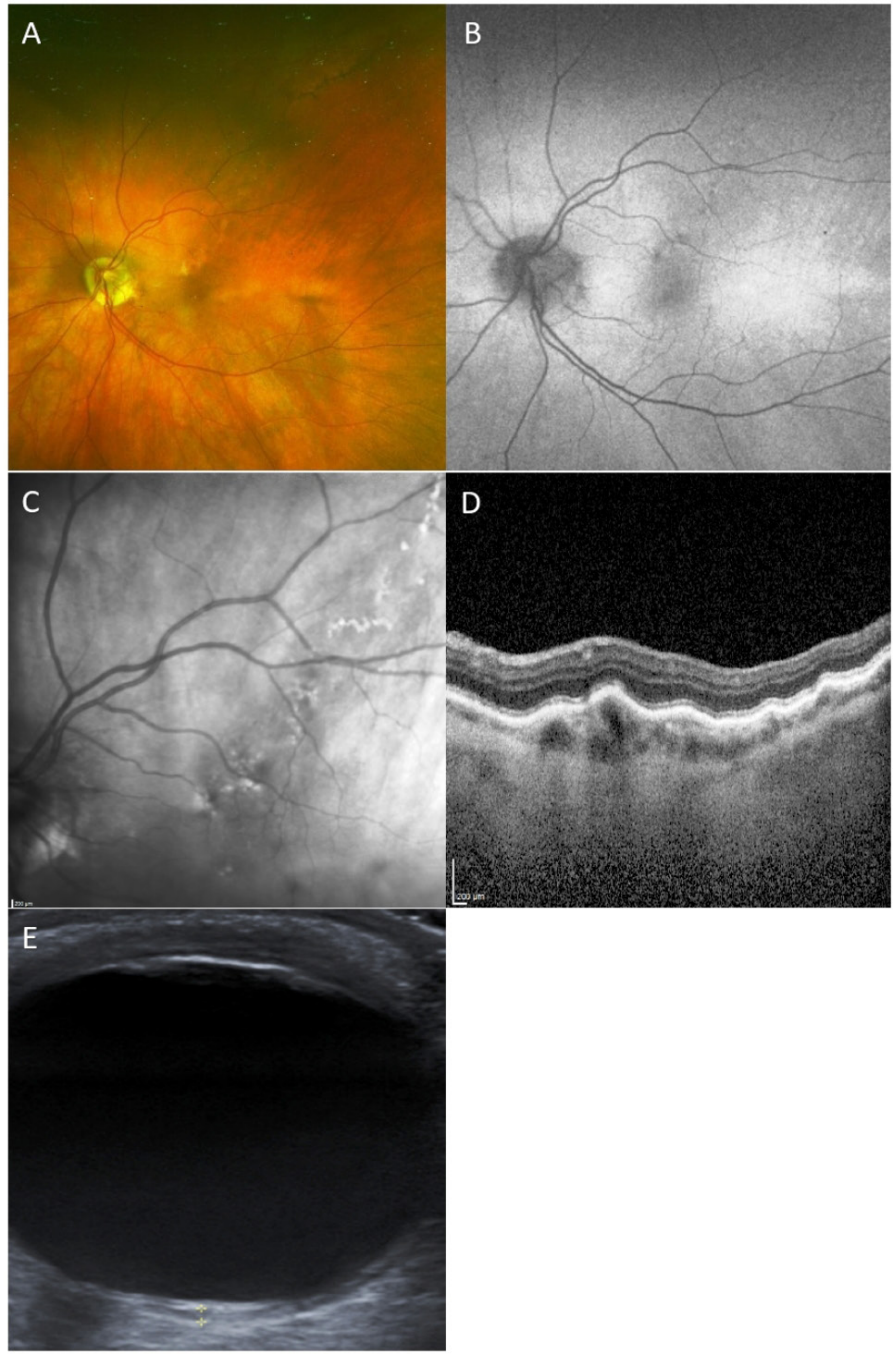
Pseudocolour fundus image, FAF, near-infrared image, EDI-OCT and B-scan us of a patient with type II CM. (A) Pseudocolour image shows RPE mottling in a track-like fashion. (B) FAF shows a hyperfluorescent and hypoautofluorescent track. (C) Near-infrared image shows hyper-reflectivity and hyporeflectivity. (D) EDI-OCT shows a hollow elevated choroidal area, occupying the entire choroid that is focally thickened. (E) On B-scan US, the CM presents as a nodular lesion with low internal echogenicity. CM, choroidal macrovessel; EDI, enhanced depth imaging; FAF, fundus autofluorescence; OCT, optical coherence tomography; RPE, retinal pigment epithelium; US, ultrasonography.

**Figure 3:**
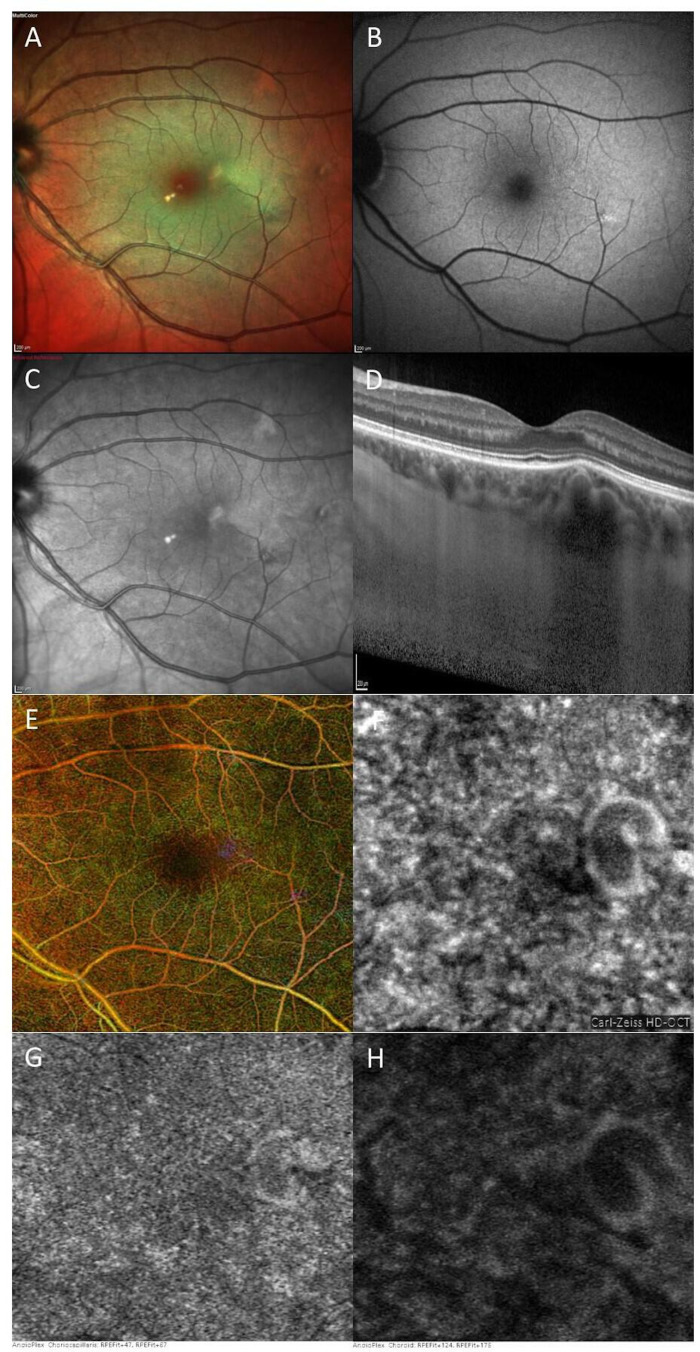
Composite multicolour, FAF, near-infrared image, EDI-OCT and OCT-A of type III CM temporal to the fovea. (A) Composite multicolour image shows mild RPE changes. (B) FAF shows tiny hyperautofluorescent spots. (C) Near-infrared image shows an area of increased reflectance. (D) EDI-OCT shows choroidal hollowness and mild elevation of the overlying retina. (E) Pseudocolour superimposition of all OCT-A scans highlights two distinguishable purple alterations. (F) structural en face choroid scan showing the CM. (G, H) Scans at the level of choriocapillaris (G) and choroid (H), both showing a vessel having a larger diameter and a hyporeflective centre. CM, choroidal macrovessel; EDI, enhanced depth imaging; FAF, fundus autofluorescence; OCT-A, optical coherence tomography-angiography; RPE, retinal pigment epithelium.

Clinically and on colour fundus photography CM had three distinct patterns (figures 1–3). In type I (6 eyes, 38%) the CM was visible as an orange-red serpiginous lesion, extending temporally from the fovea and having a tapering diameter towards the periphery. This type was easily visible funduscopically and on colour and near infrared photography (figure 1A, C). Type II (3 eyes, 19%) was characterised by retinal pigment epithelium (RPE) mottling with hyperpigmentation and hypopigmentation in a track-like fashion. The near-infrared images were more useful in detecting the CM than colour photography (figure 2A, C), with confirmation on EDI-OCT imaging (figure 2D). In type III (7 eyes, 44%) there were only spots of mild RPE changes temporal (4 eyes) or nasal (1 eye) to the fovea (figure 3A). Type III CMs were also more evident on near infrared reflectance images and confirmed on EDI-OCT (figure 3C, D).

The CM was macular in 14 eyes (88%) and extramacular in 2 eyes (12%). Macular CM extended temporally in 7 eyes (50%), superotemporally in 4 eyes (29%), superonasally in 1 eye (7%), both nasally and temporally in 1 eye (7%), both superiorly and inferiorly in 1 eye (7%). Among the 2 extramacular CMs, one was located inferotemporally to the optic disc and the other above the supero-temporal vascular arcade. The average length of the CM measured with the calliper function on OCT was 7.3 mm (median 5.55 mm; range 3.1–15.2 mm). The orientation of the CM was horizontal in 11 eyes (69%), oblique in 4 eyes (25%) and vertical in 1 eye (6%) (online supplemental figure 1). A tapering end was seen in 6 eyes (38%). Bilateral CMs had the same ophthalmoscopic appearance and orientation in both eyes, but the diameter was more prominent in one eye.

10.1136/bjophthalmol-2020-318095.supp1 Supplementary data

Enhanced depth imaging-OCT (EDI-OCT) allowed detailed choroidal imaging. The scans demonstrated the course of the lesion. In all 16 eyes (100%) the CM presented as a round or oval choroidal area with hollow reflectivity and posterior shadowing, occupying the entire thickness of the choroid and causing elevation of the overlying retina and posterior displacement of the sclerochoroidal junction (figures 1D–3D). The vessel appeared to arise predominantly from Haller’s layer. The choroidal thickness—subfoveal or extrafoveal based on the CM location—was measured with the calliper function of the OCT software and was defined as the vertical distance from the hyper-reflective line of Bruch’s membrane to the sclerochoroidal junction, and if the latter was not visible the outermost hyporeflective area of the choroid was measured.

The choroidal thickness was adjusted for the age of the subjects and the location of the CM (macular/extramacular) using reference values reported before.^13^ On OCT, CM was seen in association with choroid of normal thickness in 12 eyes (75%) from 9 patients and with thick choroid in 4 eyes (25%) from 4 patients. Of the 4 eyes with thickened choroid, the fellow eye choroidal thickness, available for 2 eyes, was normal. In bilateral cases the choroid thickness was normal.

Other OCT features included RPE irregularity in 8 eyes (50%), ellipsoid band disruption in 5 eyes (31%) or disturbance in 5 eyes (31%), subretinal fluid (SRF) in 4 eyes (25%). The SRF was macular in 3 eyes (19%) and extramacular in 1 eye (6%). Macular SRF was subfoveal in 2 eyes (13%) and extrafoveal in 1 eye (6%). The average vertical and horizontal diameters of the vessel—measured using the calliper function at the level of the ampulla of the CM when visible (14 eyes)—were 233 µm and 429 µm, respectively. OCT findings for our patient cohort are summarised in table 2.

**Table 2:** Summary of clinical characteristics of CMs on fundus photograph, EDI-OCT, FAF, ICGA, FFA, OCT-A and B-scan US

	n=16 (%)
Orientation	
Horizontal	11 (69)
Oblique	4 (25)
Vertical	1 (6)
Tapering end	6 (38)
Fundus appearance	
Type I	6 (38)
Type II	3 (19)
Type III	7 (44)
Sclero-choroidal junction compression	16 (100)
Choroid thickening	4 (25)
RPE irregularity	8 (50)
Ellipsoid band disruption	5 (31)
Ellipsoid band disturbance	5 (31)
Macular elevation	14 (88)
Sub-retinal fluid	
Macular, subfoveal	2 (13)
Macular, extrafoveal	1 (6)
Extramacular	1 (6)
Fundus autofluorescence	
Normal	6 (50)
Linear hyperfluorescence	2 (17)
Hyper/hypofluorescent track	4 (33)
ICGA	
Early filling, late staining, no leakage	6 (100)
FFA	
Early hyperfluorescence, late staining, no leakage	4 (80)
Normal	1 (20)
OCT-A	
Deep capillary plexus attenuation	1 (50)
Deep and superficial capillary plexus attenuation	1 (50)
B-scan US	
Low reflectivity nodular lesion	6 (75)
Lesion not detected	2 (25)

CM, choroidal macrovessel; EDI, enhanced depth imaging; FAF, fundus autofluorescence; FFA, fundus fluorescein angiography; ICGA, indocyanine green angiography; OCT-A, optical coherence tomography-angiography; RPE, retinal pigment epithelium; US, ultrasound.

FAF was performed in 12 eyes (10 patients) and showed no abnormalities in 6 eyes (50%), a hyperautofluorescent/hypoautofluorescent track in 4 eyes (33%) (figures 1B and 2B) and linear hyperautofluorescence in 2 eyes (17%) (figure 3B). In one case with a hyperautofluorescent/hypoautofluorescent track there was also a foveal hyperfluorescent halo corresponding to the SRF seen on OCT (figure 1B).

OCT-A is a non-invasive diagnostic tool offering a rapid three-dimensional visualisation of blood flow in retinal, choriocapillaris and choroidal circulation without injecting any contrast^14 15^ and by the segmentation in layers. The en face scans, performed in 2 eyes (2 patients), allowed visualisation of the CM at the level of the choroid and of the choriocapillaris and revealed the course through the macula, with a reflectivity similar to the surrounding vessels but a larger diameter. The CM was observed in the choroid with a disturbance of the choriocapillaris in the absence of SRF in one patient (figure 3E–H), whereas in the other patient SRF was present.

Ocular B mode US was performed in 8 eyes (seven patients) and showed in 6 eyes (75%) a nodular lesion with low internal reflectivity and an average thickness of 0.84 mm (median 0.80 mm; range 0.80–1.05 mm) (figure 2E), while in 2 eyes (25%) the CM was too shallow to be detected.

FFA performed in 5 eyes (5 patients) showed focal hyperfluorescence due to window defects in the early phase that did not increase in intensity or width in the late phases, with staining in four eyes (80%). No evidence of arterio-venous anastomosis, ischaemia or late leakage was detected. In 1 eye (20%) with extramacular CM the wide field FFA was normal.

Features on ICGA in 6 eyes of 6 patients showed hyperfluorescence of the CM and delineation of its serpiginous shape in the early phase, while in the later phases the fluorescence decreased to become similar to the surrounding vessels with focal hypofluorescent spots (figure 1E–G). Both FFA and ICGA showed no leakage even in presence of SRF. The early filling of CM on both modalities of angiography (online supplemental figure 2) suggests that it could be either an artery or a vein that carries arterialised blood flow. In 4 eyes the CM (2 of which were extramacular) showed a branching on ICGA, that was observed also on fundus photograph. Clinical findings from the different imaging techniques of all patients are summarised in table 2.

10.1136/bjophthalmol-2020-318095.supp2 Supplementary data

## Discussion

CM is a relatively rare anatomical abnormality, which can be confused with parasitic infections or even vascular fundus tumours. To date this series is the most sizeable collection of CM cases reported, with this entity mainly described in case reports in older adults. We observed a patient outside the commonly reported range, aged 27 years, and therefore an early onset or congenital nature of CM cannot be excluded. The youngest affected individual in the literature is a case reported by Turgut and Kobat in a 12-year-old with retinitis pigmentosa.^11^ In agreement with previously reported cases, CM was found in females more frequently.^2^ Caucasian and African ethnicities were found, but ethnicity was unrecorded for a number of patients. The participant numbers are too small to know whether this is an important factor or it is just easier to discern a CM in a pale fundus.

CM is considered an isolated ocular finding, since no confirmed systemic association has been found. The systemic conditions previously reported in the literature include: hypothyroidism in 2 patients,^2 8^ hyperlipidaemia in 2 patients,^2^ hypertension in 1 patient,^2^ migraine in 1 patient,^8^ chronic pancreatitis in 1 patient,^8^ urinary incontinence in 1 patient,^8^ skin basal cell carcinoma in 1 patient.^2^ The most frequently observed systemic comorbidities in our cohort were: hypertension in 5 patients (39%), diabetes mellitus in 3 patients (23%), hypothyroidism in 1 patient (8%), peripheral vascular disease in 1 patient (8%), heart disease in 1 patient (8%), hyperlipidaemia in 1 patient (8%), benign prostate hypertrophy in 1 patient (8%), vertigo in 1 patient (8%) and breast cancer in 1 patient (8%).

CM has seldom been found with other ocular disorders in the past, occurring with dry AMD in only three patients^8–10^ and macular drusen in one patient.^5^ In contrast, our cohort had a wider spectrum of ocular conditions, including glaucoma, AMD, non-proliferative diabetic retinopathy, retinal detachment, central retinal vein occlusion, branch retinal vein occlusion. It is possible that an ocular comorbidity simply makes it more likely that an asymptomatic CM is detected, rather than representing a true association.

Previous reports found only unilateral CM. In our study, we observed bilateral CMs in 23% of cases. CMs reported previously were all located within the macula, temporal to the fovea with extension towards the equator. Instead, in our cohort we observed CMs at the nasal edge of the fovea and some were extramacular. We also found CMs with an oblique and a vertical orientation. All these observations are novel, and it would be interesting to see if larger studies corroborate these.

Choudhry and Rao,^7^ Mori *et al* 12 and Dalvin *et al* 2 each reported 1 symptomatic case: the first two having metamorphopsia and dry OCT, and the second with decreased vision from SRF and ellipsoid zone alteration/disruption. In our cohort, despite observing ellipsoid zone alteration/disruption in 10 eyes, only the 2 eyes with subfoveal SRF were symptomatic. The exact pathogenic mechanism of SRF is unknown; a possible explanation is that compression exerted by the CM on the choriocapillaris leads in some cases to failure of RPE pump function. One patient was affected by concomitant nAMD, likely to contribute more to symptoms than the CM, though Inoue *et al* hypothesised that a CM may hasten AMD from relative ischaemia due to compression of the choriocapillaris or direct mechanical action on the RPE cells.^10^ Other OCT features included irregular RPE in 50% and choroid thickening in 25% of eyes. Using the OCT calliper function, we observed an average vertical diameter (233 µm) of the CM similar to that previously reported (250 µm) and a slightly shorter average length (7.3 mm vs 8.6 mm).^2^

FAF was normal in 50% of eyes with CM but other patterns were a hyperautofluorescent/hypoautofluorescent track or linear hyperautofluorescence. These findings may represent different stages of the abnormality and its local effects on the overlying RPE. Previous data about FAF are limited and included a variable hyperfluorescence over the lesion.^4 5 10^

The visualisation of choroidal vascularity on OCT-A is not always straightforward owing to the presence of imaging artefacts^16–18^: the deep choroidal vessels can be difficult to visualise due to projection artefacts from the vessels of the more superficial layers. In the first report of OCT-A in CM,^9^ it was described as a relatively low flow vessel in the choriocapillaris. On OCT-A our 2 CMs, identified at the level of both choriocapillaris and choroid, the internal flow was outside the detectable threshold, and this signal fading could relate to a too fast flow creating a fringe washout artefact.^17^ This finding, together with the early filling on ICGA suggests an arterial nature and hence a high flow of CM.

ICGA is an important tool to understand the vascular nature of the CM and helps to rule out choroidal vasculitis and other alternative diagnoses, such as a choroidal haemangioma. FFA showed non-specific findings, in line with those previously reported,^1 2 4 5 7 12^ and is of limited value for the diagnosis of CM but it helps in differentiating it from inflammatory causes.

Parasitic infestations are a frequent differential diagnosis of CM as the pigmented chorioretinal atrophic spots may be confused with residual scars from an inflammatory process. Nematode infections, especially *Oedemagena Tarandi* and *Alaria Americanus*, cause a serpiginous cicatricial track at the level of the RPE generally accompanied by exudation.^1^ Only one patient had a history of tropical travel, but intraocular inflammation was absent in all our cases. Diffuse unilateral subacute neuroretinitis has been included in the differential diagnosis of CM,^1^ but it does not produce subretinal tracks and OCT-A allows the visualisation of the worm as an avascular hyper-reflective vermiform lesion.^19 20^

Some cases are referred with a possible choroidal neoplasm, particularly circumscribed choroidal haemangioma. However, circumscribed choroidal haemangioma has different features: on OCT it has a dome-shaped configuration and circumscribed dilated choroidal vascular channels, and on ICGA has an early filling with early maximal fluorescence and a late wash-out.^21 22^ This pattern and the shape of the lesion is different to the CM.

Other vascular lesions also need to be ruled out. Vortex veins ampullae are venous in nature while CMs appear to be arterial. CMs differ from retinochoroidal anastomoses that shunt retinal venous drainage into the choroid, since they do not communicate with the retinal circulation. Moreover, none of our cases had a history of previous ocular trauma, inflammation and perifoveal teleangectasis that could have caused abnormal shunts.^23 24^ Anomalous posterior ciliary vessels provide the blood supply for the anterior uvea, unlike the CM, which extends posteriorly. Congenital retinal macrovessels can be distinguished by retinal location on OCT and delineation on FFA showing a prolonged transit time with possible ischaemia and leakage.^1 7^

The limitations of our study include the retrospective nature and the small sample cohort, hence the repeatability of our fundoscopic classification needs to be verified. There is likely to be an ascertainment bias as CMs in the posterior pole, rather than in the periphery, are more likely to be detected. We acknowledge that the measurement of the length or calibre of an irregular curved structure in three dimensions is prone to bias. OCT-A was performed in only 2 eyes, so it is unclear how far our findings would generalise to CM. No data on axial length are available. Longitudinal studies including larger numbers of patients will help overcome these barriers to better understand the epidemiology, pathogenesis, natural history and prognosis of CM. Multimodality imaging could help make more easily the diagnosis of this entity, and therefore, will become better recognised.

## Data Availability

Data are available on reasonable request. All data relevant to the study are included in the article or uploaded as online supplemental information.
