# Supplementary material for: Choroidal macrovessels: multimodal imaging findings and review of the literature
Source: Br J Ophthalmol. 2021 Jan 4;106(4):568–75. doi: 10.1136/bjophthalmol-2020-318095 (PMC8961769; doi:10.1136/bjophthalmol-2020-318095)
Supplement: Supplementary data [file bjophthalmol-2020-318095supp001.pdf]

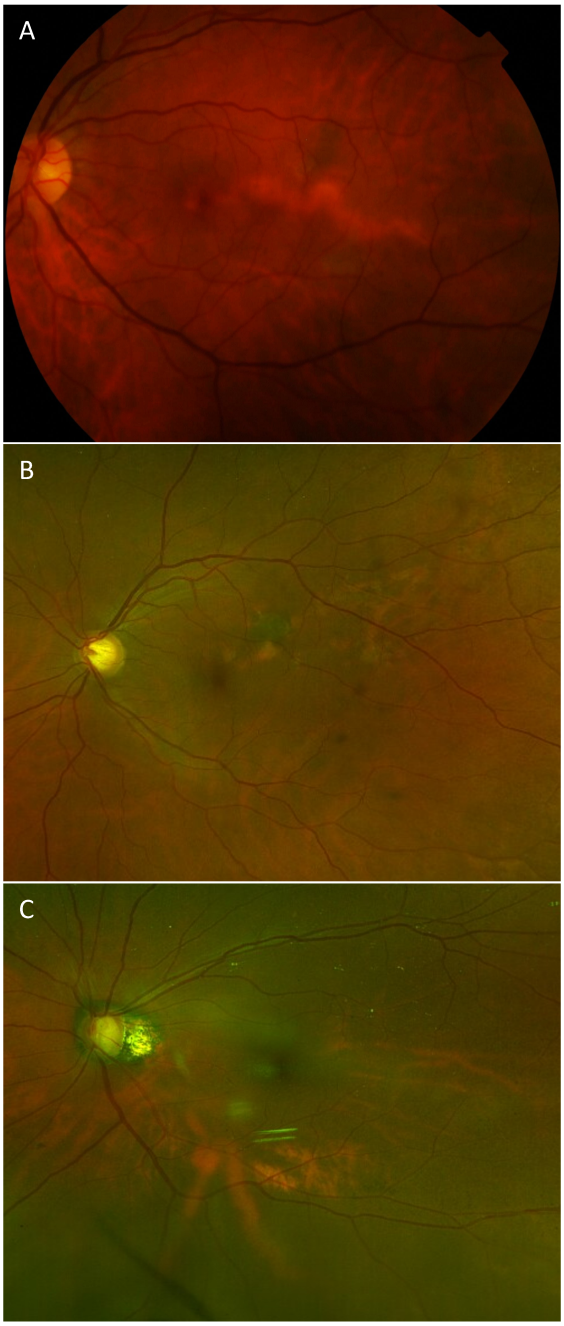

Figure I: Left eye colour fundus photographs showing three different patterns of orientation of CM. A: CM with horizontal orientation extending temporally to the fovea with a tapering diameter. B: oblique orientation of a CM extending supero-temporally to the fovea. C: extra-macular branched CM with vertical orientation, located infero-temporally to the optic disc.
