# Supplementary material for: Choroidal macrovessels: multimodal imaging findings and review of the literature
Source: Br J Ophthalmol. 2021 Jan 4;106(4):568–75. doi: 10.1136/bjophthalmol-2020-318095 (PMC8961769; doi:10.1136/bjophthalmol-2020-318095)
Supplement: Supplementary data [file bjophthalmol-2020-318095supp002.pdf]

BMJ Publishing Group Limited (BMJ) disclaims all liability and responsibility arising from any reliance placed on this supplemental material which has been supplied by the author(s)

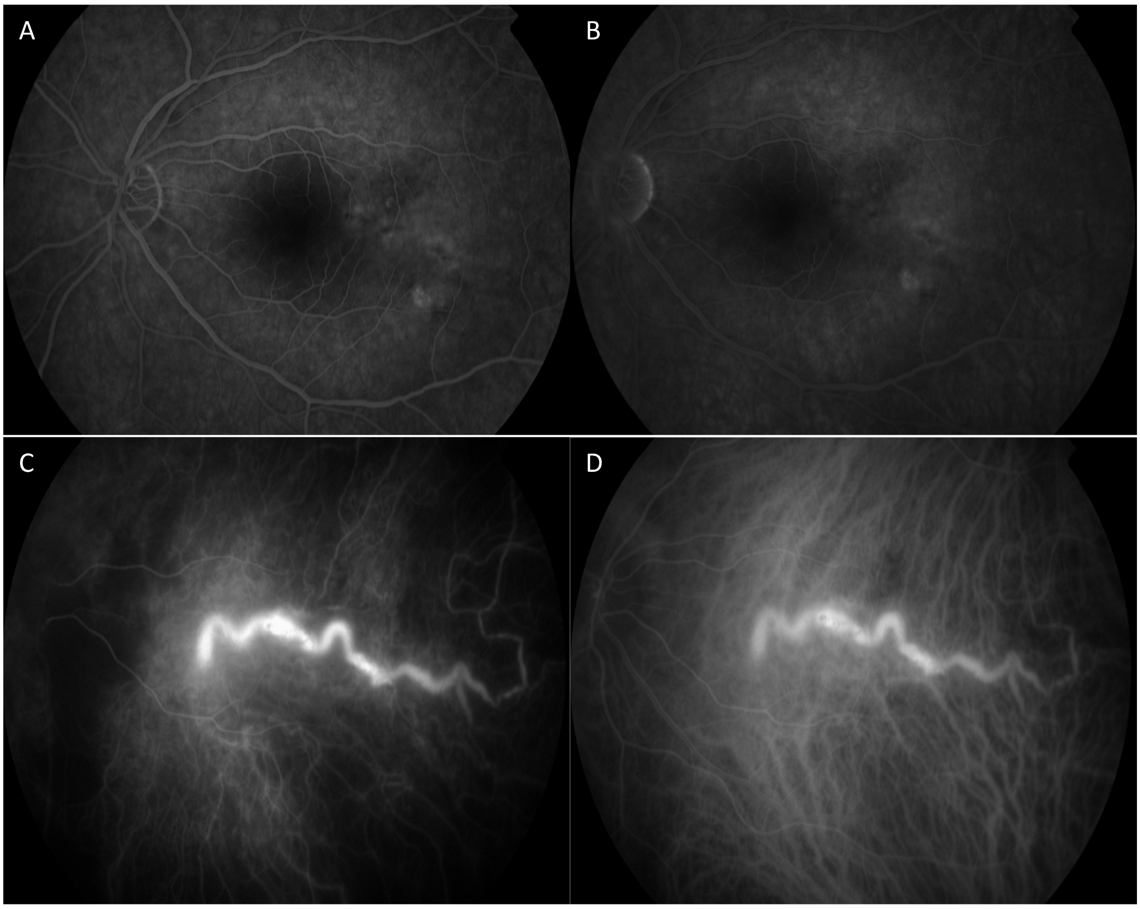

Figure II: Left eye FFA and ICGA of a patient with macular CM. A-B: on FFA the CM presents an early focal hyperfluorescence, due to window defects, that in the late phases does not increase in intensity or width and shows staining. C-D: on ICGA the CM has an early lighting up and a serpiginous shape; in the later phases the fluorescence decreases and focal spots of hypofluorescence along the vessel become visible.
